# Costly conspicuousness reveals benefits of sexual dimorphism in brood parasitic diederik cuckoos

**DOI:** 10.1002/ece3.11263

**Published:** 2024-05-21

**Authors:** Jennifer York

**Affiliations:** ^1^ Department of Zoology University of Cambridge Cambridge UK; ^2^ Department of Zoology and Entomology University of Pretoria Hatfield, Pretoria South Africa; ^3^ Department of Ecology and Evolutionary Biology Princeton University Princeton New Jersey USA

**Keywords:** brood parasitism, coevolution, Cuculidae, discrimination, sexual dimorphism

## Abstract

The existence of adult sexual dimorphism is typically explained as a consequence of sexual selection, yet coevolutionary drivers of sexual dimorphism frequently remain untested. Here, I investigate the role of sexual dimorphism in host–parasite interactions of the brood parasitic diederik cuckoo, *Chrysococcyx caprius*. Female diederik cuckoos are more cryptic in appearance and pose a threat to the clutch, while male diederik cuckoos are conspicuous and not a direct threat. Specifically, I examine whether sexual dimorphism in diederik cuckoos provokes threat‐level sensitive responses in Southern red bishop, *Euplectes orix*, hosts. I use experimentally simulated nest intrusions to test whether hosts have the capacity to differentially (i) detect, and/or (ii) discriminate between, male and female diederik cuckoos, relative to harmless controls. Overall, I found no evidence that diederik cuckoos differ in detectability, since both sexes are comparable to controls in the probability and speed of host detection. Furthermore, neither male nor female hosts discriminate between sexually dimorphic diederik cuckoos when engaging in frontline nest defences. However, hosts that witnessed a male diederik cuckoo during the trial were more likely to reject odd eggs. Moreover, experimental eggs were significantly more likely to be rejected when female bishops observed a male compared to a female diederik cuckoo. While the cryptic appearance of female diederik cuckoos does not reduce detection by hosts, it does provide the benefit of anonymity given the egg rejection costs of conspicuous male‐like appearance in the nest vicinity. These findings have implications for the evolution and maintenance of sexual dimorphism across the Cuculidae, and highlight the value of testing assumptions about the ecological drivers of sexual dimorphism.

## Graphical abstract

Sexual dimorphism is typically explained by sexual selection, yet other potential drivers frequently remain untested. I examine whether Southern red bishop, *Euplectes orix*, hosts of the brood parasitic diederik cuckoo, *Chrysococcyx caprius,* differentially detect, and/or discriminate between, the male and female forms of their brood parasite. Using experimental intrusions at hosts’ nests, I found no evidence that diederik cuckoos differ in detectability, and hosts were also indiscriminately aggressive toward nest intruders. However, hosts that witnessed a male diederik cuckoo model during the trial were more likely to reject odd eggs. Together, these findings suggest that the more cryptic appearance of female diederik cuckoos is beneficial given the egg rejection costs associated with conspicuous male‐like intruders at the nest. The female diederik cuckoo (left) exhibits less conspicuous plumage and facial colouration than the male (right; photography by Dominic Cram and Jenny York).

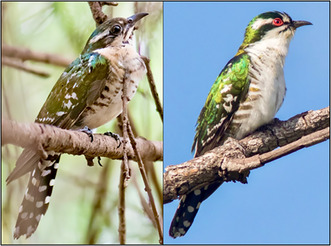

## 1 INTRODUCTION

Sex differences in adult phenotypes can arise because of dissimilar selection acting on the sexes (Andersson, 1994; Darwin, 1871; Mank, 2008; Parker et al., 1972). While evidence supports the role of sexual selection as an underlying driver of sexually dimorphic phenotypes in many cases, the contribution of natural selection frequently remains untested, despite evidence that multiple selective pressures can contribute to sex‐specific adult phenotypes (Law & Mehta, 2018; Owens & Hartley, 1998; Shine, 1989; Székely et al., 2000). Consequently, it is valuable to examine the ecological drivers and adaptive value of sex‐linked traits (Runemark et al., 2018; Shine, 1989).

Brood parasitic cuckoos (Cuculidae) provide an interesting test case in the evolution of adult sex differences for three main reasons. First, unlike 99% of birds, neither sex of brood parasitic cuckoo rears their own young and, consequently, there is also no differential mating success due to selection for indirect benefits of early‐life care that can arise in parental species (Kokko & Jennions, 2008; Krüger, 2007; Payne, 1967; Trivers, 1972). Second, comparative analyses of sexual dimorphism in cuckoos show that sexual selection, which typically selects for larger body size in males, does not do so in brood parasitic cuckoos (Krüger et al., 2007). Third, sexual dimorphism instead arises via female‐biased phenotypic change in brood parasitic compared to parental cuckoos (Krüger et al., 2007). Crucially, across the Cuculidae, the brood parasitic cuckoo females are more cryptic than males and this sexually dimorphic crypsis is likely important in females avoiding detection by hosts (Krüger et al., 2007; Payne, 1967). However, whether the hosts of brood parasitic cuckoos differentially detect, and/or discriminate between, adult forms due to sex differences in characteristics has rarely been investigated (York & Davies, 2017). This is important because identifying brood parasitic threats allows hosts to mount behavioural defences such as aggressive mobbing and egg rejection, which can be costly or fatal for cuckoos, and are key to mechanisms for selection on adult brood parasitic cuckoo phenotypes (reviewed in York, 2021).

**FIGURE 1:**
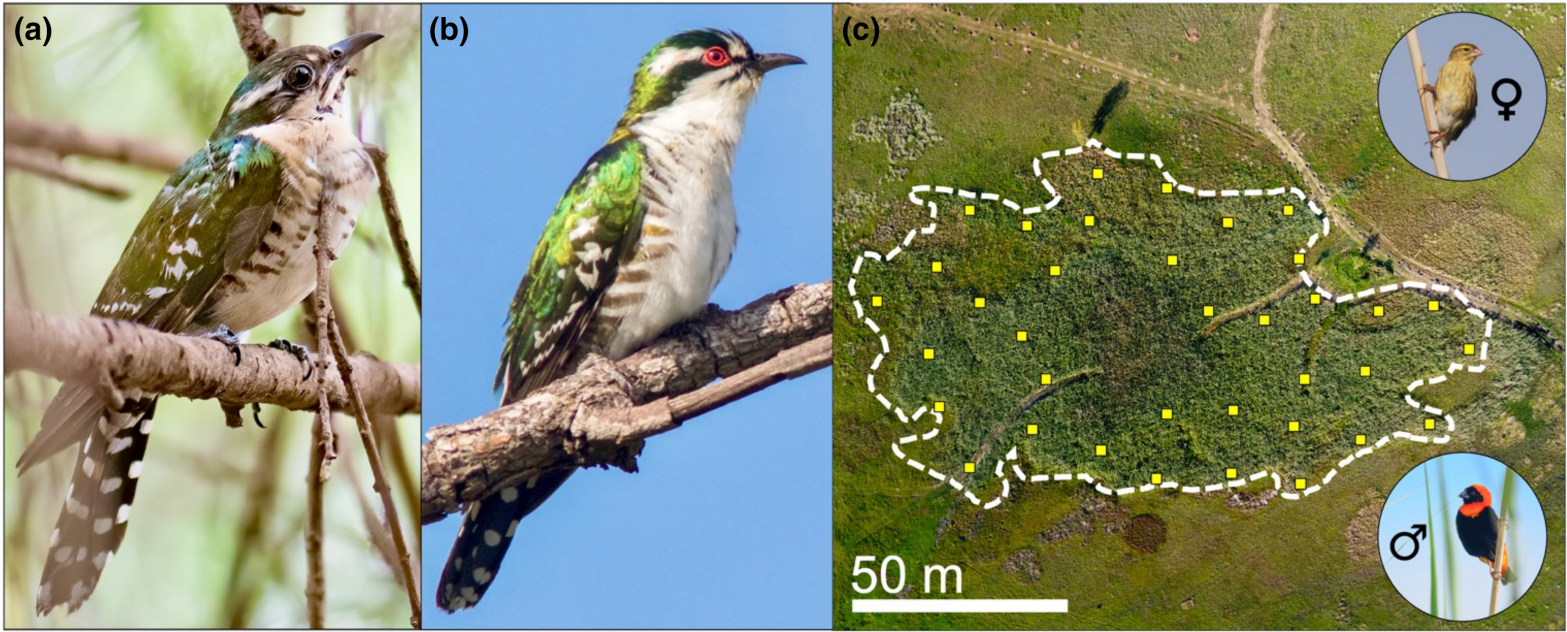
The sexually dimorphic diederik cuckoo, *Chrysococcyx caprius*, (a) female (plumage duller, and the iris and eye‐ring are brown), and (b) the more conspicuous male (breast plumage bright white, with vibrant red iris and eye‐ring) and (c) an aerial view of a Southern red bishop, *Euplectes orix*, colony indicating the distribution of experimental nest territories (yellow squares represent territory area); inset female bishop (top right) and male bishop (bottom right; all photographs by D. Cram and J. York).

Here, I test whether host defences against brood parasitism differ according to sex differences in adult diederik cuckoo (*Chrysococcyx caprius*) appearance using a model presentation experiment at the nests of free‐living hosts. Diederik cuckoos are sexually dimorphic in plumage and facial colouration, with females presenting a more cryptic adult phenotype than conspicuous males, while both sexes are similar in size (Figure 1; Krüger et al., 2007; Payne, 1967, 2005; Reed, 1968; Rowan, 1983). They are obligate, host‐evicting brood parasites with host species including the Ploceidae (the weaverbirds; Payne, 2005; Rowan, 1983). One species regarded a particularly frequent host is the Southern red bishop (*Euplectes orix*; hereafter: ‘bishop’), but remarkably little is known about the ecological and evolutionary dynamics between bishops and diederik cuckoos (Lawes & Kirkman, 1996; Reed, 1968; Rowan, 1983). Bishops are polygynous and colonial weaverbirds that occur widely across sub‐Saharan Africa (Friedl, 2004; Friedl & Klump, 1999; Lawes et al., 2002; Metz et al., 2009). In wetland habitat dominated by Phragmites reeds, male bishops defend small (typically 3 m across) breeding territories where they build numerous nests to attract females (Metz et al., 2009). Upon selecting a nest, the female bishop lays her eggs, then incubates and provides care for offspring, which can include a brood parasitic diederik cuckoo chick. Brood parasitism incidence varies widely and ranges from 0% to 67% of nests across colonies at different sites and between years (Hunter, 1961; Jensen & Vernon, 1970; Payne & Payne, 1967; Rowan, 1983).

Specifically, I examine whether the sexual dimorphism of male and female diederik cuckoos provokes threat‐level sensitive responses in bishops. First, I test the hypothesis that the cryptic appearance of female diederik cuckoos has evolved due to the benefits of being less detectable to hosts. Using simulated intrusions of male and female diederik cuckoo at the host's nest, I test whether males and females differ in detectability (probability and speed) by their hosts, relative to sexually monomorphic, harmless controls (dark‐capped bulbuls, *Pycnonotus tricolor*). Given the evidence that, across species, brood parasitic females are more cryptic (Krüger et al., 2007; Payne, 1967; Reed, 1968), I predict that female diederik cuckoos are less easily detected by hosts, and therefore hosts should be less likely to respond, or take longer to respond, to females compared to more conspicuous males and controls. Second, I used simulated nest intrusions to test whether hosts can discriminate between males and females, relative to controls, by exhibiting differences in behavioural defences (frontline aggressive defence and egg rejection) towards the male (no direct threat) compared to the female (high threat). The capacity to adjust behavioural defences towards intruders according to the scale of the threat they pose is observed among some species of weaverbirds (York et al., 2019). Moreover, weaverbird hosts are aggressive towards diederik cuckoo, and while they will readily strike diederik cuckoo mounts, they produce milder aggression towards other species of cuckoo that do not target weaverbirds as their hosts, which suggests an underlying capacity to discriminate between heterospecifics in accordance with the threat they present (Lawes & Kirkman, 1996; Noble, 1995; Rowan, 1983). I predict that if hosts do discriminate, they would benefit from directing greater aggression and stronger egg rejection defences towards the greater brood parasitic threat of female diederik cuckoos. Finally, given that host populations are heterogeneous in terms of the defences that brood parasites experience on approaching a nest, I examine the role of intraspecific variation in host responses to intrusions at the nest by quantifying whether male and female hosts differ in their responses to the simulated intrusions. I predict that male hosts will be more aggressive towards the intruding threat than females because males build and defend nest structures, so they are likely to be more vigilant and aggressive towards intruders in the nest vicinity.

## 2 MATERIALS AND METHODS

### 2.1 General methods

I conducted fieldwork between October 2019 and March 2020 and collected data for this experiment from a population of diederik cuckoo and bishops on private wetlands in Gauteng, South Africa, where I have been observing and studying these species since circa 2017. Each year, breeding bishop males build multiple nests on small territories (typically 3 m across) to attract females (Figure 1c shows a section of reed bed). Nests were monitored from construction through laying and incubation using individual markers on a supporting reed stem. Nest locations and placement were monitored and male movements between nests were observed with binoculars. Diederik cuckoos are intra‐African breeding migrants that arrive in the highveld region from the first weeks of October onwards, with peak laying activity in December (Reed, 1968). The onomatopoeic common name ‘diederik’ comes from the distinctive whistling ‘dee dee dee dee‐derik’ call of the male which is broadcast loudly and frequently during the breeding season (Reed, 1968). Males at this site were heard calling and seen displaying throughout the study period. Interspecific brood parasitism was assessed by observing egg size and appearance and whether a pencil mark adhered to the shell (Lawes & Kirkman, 1996; Lindholm, 1997). Natural brood parasitism incidence in this population during the study period occurred in 7%–20% (87 monitored to clutch completion) of nests, with 7% matching diederik cuckoo egg characteristics and 20% including potential intra‐specific brood parasitism (Lawes & Kirkman, 1996; Lindholm, 1997). These estimates were not confirmed with nestling characteristics in the majority of cases. Natural brood parasitism events are brief and challenging to study, and all data presented here use the experimental approach described below.

### 2.2 Experimental design

I conducted an independent‐measures experiment with 72 host subjects at 36 nests between December 2019 and January 2020 on days when weather conditions were dry and wind levels were low. At each nest, I simulated a brood parasitism attempt by a diederik cuckoo following previous methods (Davies & Brooke, 1988; Thorogood & Davies, 2016; York & Davies, 2017). Because diederik cuckoos remove one egg from the clutch before replacing it with their own (Reed, 1968), immediately before starting each trial I simulated this in the field by selecting one egg at random from the clutch at each nest and painting it with a layer of Mont Marte acrylic ‘titanium’ white, dotted at random with ‘burnt umber’ brown spots, before returning the air‐dried, painted egg to the nest. This approach facilitates studies of egg rejection by hole ejectors (they peck a small hole in the shell to grip the egg and eject it from the nest) and eggs that are not rejected by the host will subsequently hatch (Thorogood & Davies, 2016; York & Davies, 2017). In several cases where the nests were over dry ground, the experimental painted egg was found below the nest with a small hole pecked in the shell due to host ejection. I used a heavily maculated non‐mimetic egg appearance similar to the Southern masked weaver (*Ploceus velatus*) because (1) this pattern is similar to the eggs laid by some diederik cuckoo at this site, and cuckoos will occasionally lay in the nest of non‐preferred hosts (Davies, 2000), (2) this host species is not highly discriminating towards model eggs during egg laying or after clutch completion, but will reject heavily maculated or greatly mis‐matched model eggs across this period (Lawes & Kirkman, 1996) and (3) rejection rates in this population were previously unknown, so a non‐mimetic egg ensured interpretable data regardless of how discriminating hosts were against egg appearance. Immediately following the brood parasitism simulation in the clutch, I positioned an adult bird model (details below) on the outside of the nest at the lip of the entrance hole and positioned a video camera (Panasonic HC‐V270EB‐K HD) on a tripod at 5 m from the focal nest, started the recording and retreated to observe the focal nest with binoculars from a distance of at least 20 m. After the trial, I returned to collect the camera and remove the model. In all cases, hosts were observed in the reeds surrounding the focal nest area during the experimental trial.

The model type presented at each nest was pre‐determined using latin square to allocate the treatments through the course of the experiment, and an independent measures design was used to avoid carry‐over effects of model presentations on the hosts, since these can elicit intense behavioural responses and lasting physiological effects which may influence subsequent behavioural responses to stimuli (Apfelbeck et al., 2011). This design also facilitated measurement of egg rejection responses through simulated brood parasitism, which can only be carried out once per nest after exposure to adult heterospecific model stimuli. I alternated between two identical model exemplars of each of the three treatment types. Nests were sufficiently separated (at least 10 m from the nearest experimental territory, and separated by non‐experimental neighbouring territories between, each typically 3 m across in reedbed habitat; Figure 1c) to avoid model presentations at one nest influencing responses at another. Treatments were distributed across the population nesting area each day and territories were not selected in the most central region of the reed bed to mitigate positional effects on host responsiveness that could conceivably arise through nesting density (Ferguson, 1994; Lawes & Kirkman, 1996). Trials were temporally distributed on the same day (typically 40 min between), and across days through the experimental period (December–January). Simulated intrusions were carried out during morning or afternoon, when natural brood parasitism attempts are more likely to occur (Chance, 1940; Lindholm, 1997). Nest contents were checked 1 day and 3 days after the trial to record the rejection or acceptance of experimental eggs. Methodology received ethical clearance from the University of Cambridge (ZOO69/19) and the University of Pretoria (NAS197), and fieldwork was conducted under permit.

### 2.3 Model bird stimuli

The adult bird models were manufactured by an independent South African 3D model printing and finishing service that produces African bird models for research and conservation applications. The 3D models were printed (Creality CR‐10) in modified Polylactic Acid (HPLA, Shenzen Hello 3D Technology) and then finished by adding a coating of plastic primer (Car System), coloured acrylic paint (Winsor and Newton Galleria) and sealed with a polyurethane‐based concrete sealant (Duram). I presented the three model treatments (*n* = 12 nests per treatment group) with one treatment type per nest: female diederik cuckoo model (dimensions: 19.4 × 5.8 × 4.9 cm; Figure 1a), male diederik cuckoo model (19.4 × 5.8 × 4.9 cm; Figure 1b) and dark‐capped bulbul model (20.0 × 5.4 × 6.1 cm). I selected dark‐capped bulbuls as a control for four reasons: (1) they are a similar body size to diederik cuckoo (19–20 cm in length) which controls for size effects on detectability, (2) dark‐capped bulbuls are common at this site, which mitigates issues of neophobic responses to novel stimuli, (3) bulbuls present no ecological threat to bishops since they are neither predatory nor are they niche competitors and (4) although bulbul sexes are indistinguishable in their plumage colouration (so only one model type was used), their plumage includes both inconspicuous, background‐matching (drab brown) and conspicuous, background‐contrasting (bright yellow patch) colouration, which provides scope to draw comparison with the conspicuous male diederik cuckoos (bright red and white patches of background‐contrasting colouration). Since background contrast can be an important factor in stimulus detectability (Koskepato et al., 2020; Troscianko et al., 2016), the bulbul control treatment provides the capacity to interpret responses in the context of model conspicuousness and detectability (i.e. whether the model has a conspicuous background‐contrasting patch or not) versus model identity (the brood parasitism threat that the class of model presents). None of the models were finished with iridescent colouration to standardise the model design across the treatments, and because the iridescence of diederik cuckoos does not show strongly in the light environment where their hosts nest (Reed, 1968).

### 2.4 Behavioural responses

To investigate bishops' behavioural responses to model stimuli, data were collected from matched pairs of male and female individuals in the nest vicinity (the immediate area around the nest, where the focal bird was visible in the camera frame in a similar plane to the model, gauged by relative body size). Male and female bishops are conspicuously dimorphic in plumage colouration during the breeding season. During the experimental period (December 2019 and January 2020) all males were in full breeding plumage, with bright red and black colouration, which ensured ease of discriminating them from female (brown and streaky) hosts. Female bishops can also be differentiated from other locally occurring weaverbird species using relative size, plumage and body shape characteristics, and since they occur infrequently in the nest vicinity. Host behavioural responses were extracted from the 5 min trial from the videos (as below). These responses were selected to quantify (1) model detection (the probability and latency to approach the nest vicinity, and the probability and latency to approach the model), and (2) discrimination between models (the probability and latency to attack the model and the probability of egg rejection). The 5 min trial period commenced following the placement of the model and video camera. Hosts were confirmed to be present in the nest area during the trial in all cases. Because this host species nests at high density, aggressive responses by the focal hosts could (infrequently) elicit mobbing behaviour from neighbouring (non‐experimental) males and females, so experimental nests were distributed with several non‐experimental nests in‐between (Figure 1c). The behavioural responses use the initial behavioural state change (i.e. host presence, approach, strike).

Egg rejection responses were assessed at 1 day, and again after 3 days, since the model presentation trial, because cuckoo egg rejections typically occur during the first day, and relatively few occur after 3 days, and because excess nest visits can increase predation risk or clutch abandonment (Brooke & Davies, 1988; Reed, 1968). Responses were recorded for the presence (acceptance = 1) or absence (rejection = 0) of the experimental egg in the nest by examining the contents. Nests were checked for signs of depredation and in one case, nest contents were depredated at Day 1 (the nest and supporting reeds were destroyed from below). Between 24 and 26 December 2019, access to the field site was not possible and consequently some data were missing. On Day 1, there were four missing values due to three nests not being checked (one of each treatment) and one depredation (male treatment). On Day 3, there were five missing values, comprising the four missing values from Day 1, plus one additional nest not checked (male treatment). Of the 12 nests allocated to the three treatment groups (*n* = 36 nests), on Day 1 rejection data were collected from 11, 10 and 11 nests (bulbul, male diederik and female diederik groups respectively; *n* = 32 nests). On Day 3, rejection data were collected from 11, 9 and 11 nests (bulbul, male diederik and female diederik groups respectively; *n* = 31 nests). Missing data values are coded as ‘NA’.

### 2.5 Video analysis

Behavioural responses during the trial period were recorded on video at 50 frames per second, at a resolution of 1920 x 1080, with the framing and zoom view for each nest specified using the ‘grid’ function and saved as MPEG‐4 video files. The following behavioural event data were extracted for male and female bishops during the 5 min trial from the videos with VLC (VideoLan) using x0.25 playback speed to determine the following event timing of the behavioural response to the second: (i) entering the nest vicinity, where the focal bird was in a similar plane to the model (gauged by relative body size); (ii) approaching the model, where the focal bird was less than two model lengths (approximately 40 cm, within the supporting or adjacent reeds to the nest) away from the model, and had moved towards the model; (iii) first physical contact with the model: ‘strike’, using the beak or feet.

For both male and female hosts, these raw data were then used to calculate the latencies (in seconds) to: (1) entering the nest vicinity; (2) approaching the model; (3) attacking the model. All three variables were extracted for the first male and first female to enter the frame. I also calculated the lag (seconds) between the time point at which individual hosts that enter the nest vicinity and then subsequently approached the model. This ‘approach window’ was used to investigate whether the window of time between initially detecting the model (entering the vicinity) and responding to the model (approaching the model) differed across the three treatments since rapid responses on detecting stimuli are associated with aggression (Apfelbeck et al., 2011) and could be a selective pressure on laying speed among the Cuculini (Chance, 1940). In a minority of cases, additional neighbouring (non‐experimental) males and females later entered the frame to contribute to collective mobbing attacks on the model (see Section 3; Video 1).

**VIDEO 1:** A simulated male diederik cuckoo intrusion at Southern red bishop host nest with the host male attacking the model diederik cuckoo.

### 2.6 Statistical analyses

Supporting data and code are available: https://doi.org/10.5061/dryad.g79cnp5xx. All analyses were conducted in R (version 4.2.3; R Development Core Team, 2015) by fitting models with all terms of interest (the full model) and determining the significance of each explanatory variable by removing the term from the full model to test for a change in deviance in the fit of the model without that specific term (Forstmeier & Schielzeth, 2011). Linear mixed effects models (LMM, package ‘lme4’; Bates et al., 2014) and generalised linear mixed‐effects models (GLMM) were inspected for over‐dispersion, zero‐inflation, normality and heteroscedasticity, as appropriate, and were satisfactory unless otherwise stated (R package ‘DHARMa’; Hartig, 2022). The details for each analysis are provided below.

### 2.7 Frontline aggression responses

To analyse the probability that hosts respond to the simulated intrusion at their nest, I used a GLMM with binomial error (logit‐link function for each binary response term: Table 1a–c). In each case, the fixed terms ‘treatment’ (‘bulbul’, ‘male diederik’, ‘female diederik’), host sex (male, female) and the interaction between ‘treatment’ × ‘host sex’, were specified in the full model, as was the random term ‘nest ID’ to control for matched data from male and female host parents from the same focal nest. To investigate whether the ‘approach window’ of time between initially detecting the model (entering the vicinity) and responding to the model (approaching the model) differed across the three treatments, I used an LMM with a Gaussian distribution. Again, the fixed terms ‘treatment’ (‘bulbul’, ‘male diederik’, ‘female diederik’), host sex (male, female), the interaction between ‘treatment’ × ‘host sex’ and the random term ‘nest ID’ were specified in the full model. The response variable ‘approach window’ was square‐root transformed prior to analysis for normality of residuals.

**TABLE 1:** Generalised linear mixed‐effects models (GLMMs) for host behavioural responses to simulated brood parasitism: (a) probability of a male or female host entering the nest vicinity; (b) probability of a male or female host approaching the model; (c) probability of a male or female host attacking the model.

Response term	Explanatory terms	Mean ± SE	*x* ^2^	*p*
(a) Probability of host entering nest vicinity (yes/no)	Sex (male) × treatment (male)	5.63 ± 5.11	1.44	.49
	Treatment (male)	−4.86 ± 3.09	0.12	.94
	Host sex (male)	2.11 ± 2.15	1.35	.24
	*Intercept*	8.02 ± 2.93	–	–
	*nest ID* ^a^	111.6 ± 10.56	–	–
(b) Probability of host approaching model (yes/no)	Sex (male) × treatment (male)	−2.54 ± 1.58	3.04	.22
	Treatment (male)	2.00 ± 1.28	0.68	.71
	Host sex (male)	1.51 ± 1.10	0.31	.58
	*Intercept*	−0.49 ± 0.83	–	–
	*nest ID* ^a^	1.99 ± 1.41	–	–
(c) Probability of host attacking model (yes/no)	Sex (male) × treatment (male)	268.55 ± 281.34	0.13	.94
	Treatment (male)	−267.81 ± 281.33	0.01	.99
	**Host sex (male)**	**14.67 ± 8.37**	**22.78**	**<.001**
	*Intercept*	−27.72 ± 10.78	–	–
	*nest ID* ^a^	4545 ± 67.41	–	–

*Note*: The *p* values for each term are from the chi‐squared test (likelihood ratio test) for change in deviance when comparing models with or without the term (terms that resulted in a significant change in deviance when removed are bold). The untransformed estimates (logit‐link) ± standard error (SE) are reported for all terms in the full model.

^a^ Variance and SD are reported for the random term ‘nest ID’.

To analyse the latencies of aggression responses to a simulated intrusion at their nest, I used an analytical approach designed for censored data. In this experiment, all response latencies were capped at the end of the simulated intrusion trial, which was standardised to 5 min. In most cases, the responses occurred within the trial period, but where the behavioural event did not occur within the trial period, the response was allocated the maximum value of the trial duration (300 s). Consequently, for these censored data (the absolute value is constrained by the sampling approach) where the relative position of the data point is nevertheless informative (e.g. yet to respond at 5 min after the trial had begun), can be captured in the analysis. Mixed‐effects survival models (MESM) with Cox proportional hazards (Therneau, 2015; package ‘coxme’) were used because, in addition to being designed for censored data, they also permit random terms to be fitted, in this case, to control for multiple data points from the same focal nest. One model was fitted for each response term: (1) ‘latency to enter nest vicinity’; (2) ‘latency to approach the model’ and (3) ‘latency to attack the model’. In all cases, the fixed terms ‘treatment’ (‘bulbul’, ‘male diederik’, ‘female diederik’), host sex (male, female), the interaction between ‘treatment’ × ‘host sex’ and the random term ‘nest ID’ was specified in the full model.

### 2.8 Experimental brood parasitism egg rejection responses

For analyses of the probability of experimental egg rejection of hosts, I used generalised linear models (GLM) with binomial error (logit‐link function) for each binary response term (Tables 2 and 3). In each case, the fixed terms ‘treatment’ (‘bulbul’, ‘male diederik’, ‘female diederik’), whether or not the focal host male or female individual entered the nest vicinity during the trial (‘in vicinity’, yes, no), and the interaction between ‘treatment’ × ‘in vicinity’, were specified in the full model. The term ‘in vicinity’ was included because the sight of a cuckoo at the nest is known to increase the probability of hosts rejecting experimental eggs (Davies & Brooke, 1989; Thorogood & Davies, 2016). Because individual‐level egg rejection response data for each male and female host was not feasible to collect for this study (in contrast to individual‐level data on whether the host observed the model at the nest, see above), and because it was not established whether either the male or the female host is solely responsible for egg rejection decisions, a dataset was analysed for each host sex: ‘male host in vicinity during trial’ (Table 2) and ‘female host in vicinity during trial’ (Table 3), and separate analyses for each dataset are presented. Significant interaction terms were further examined by comparing the model with all three levels with a simpler model where the two levels for the contrast of interest were collapsed to test for a change in deviance in the fit of the model (i.e. with or without the level of interest defined).

**TABLE 2:** Generalised linear models (GLMs) for host egg rejection responses to simulated brood parasitism in relation to whether the *male* host did/did not enter the nest vicinity during the trial.

Response term	Explanatory terms	Mean ± SE	*x* ^2^	*p*
(a) Egg rejection within *1 day* of the trial	**Male in vicinity × treatment (male)**	**−3.91 ± 7.99**	**7.03**	**.030**
	Treatment (male)	3.71 ± 7.99	1.72	.42
	Male in vicinity	1.99 ± 4.61	2.66	.10
	*Intercept*	−1.86 ± 4.61	–	–
(b) Egg rejection within *3 days* of the trial	**Male in vicinity × treatment (male)**	**−3.90 ± 7.99**	**6.76**	**.034**
	Treatment (male)	3.71 ± 7.99	1.32	.52
	Male in vicinity	1.92 ± 4.61	1.46	.23
	*Intercept*	−1.86 ± 4.61	–	–

*Note*: The *p* Value for each term is from the chi‐squared test (likelihood ratio test) for change in deviance when comparing models with or without the term of interest (terms that resulted in a significant change in deviance when removed are bold). The mean estimates ± standard error (SE) are reported for all terms in the full model. Model estimates are untransformed (logit‐link).

**TABLE 3:** Generalised linear models (GLMs) for host egg rejection responses to simulated brood parasitism in relation to whether the *female* host did/did not enter the nest vicinity during the trial.

Response term	Explanatory terms	Mean ± SE	*x* ^2^	p
(a) Egg rejection within *1 day* of the trial	Female in vicinity × treatment (male)	−1.93 ± 2.80	4.66	.097
	Treatment (male)	1.76 ± 2.80	1.38	.50
	Female in vicinity	6.93 ± 1.58	0.24	.62
	*Intercept*	4.66 ± 1.41	–	–
(b) Egg rejection within *3 days* of the trial	**Female in vicinity** × **treatment (male)**	**−2.08** ± **4.61**	**9.05**	**.011**
	Treatment (male)	1.86 ± 4.61	1.14	.56
	Female in vicinity	2.23 ± 1.56	0.02	.89
	*Intercept*	6.88 ± 1.41	–	–

*Note*: The *p* value for each term is from the chi‐squared test (likelihood ratio test) for change in deviance when comparing models with or without the term of interest (terms that resulted in a significant change in deviance when removed are bold). The mean estimates ± standard error (SE) are reported for all terms in the full model. Model estimates are untransformed (logit‐link).

## 3 RESULTS

Male and female bishop (*n* = 72) responses during simulated heterospecific intrusion trials at host nests (*n* = 36) were qualitatively similar to those described for taxidermy mounts (Noble, 1995; Rowan, 1983) and natural interactions (pers. obs. J.E.York) in other contexts. During all trials, I observed through binoculars at a distance whether hosts were near the nest (<2 m), and in each case, this was confirmed.

### 3.1 Do male or female bishop hosts differentially detect diederik cuckoos at the nest due to sex differences in appearance?

**FIGURE 2:**
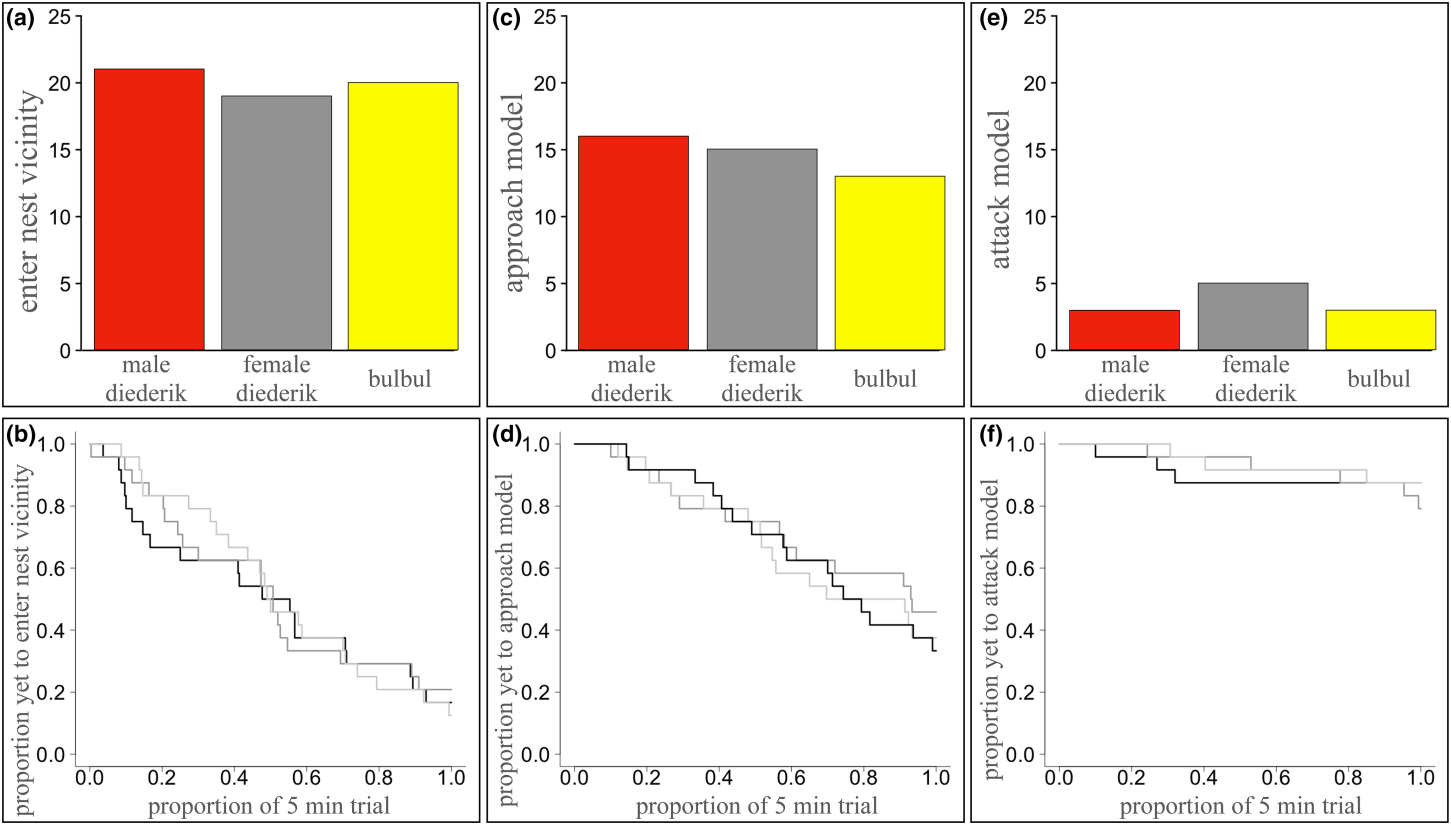
Frequencies of hosts engaging in frontline responses during the trial (top row) and relative frontline response latencies (bottom row) during simulated intrusions of heterospecifics at hosts' nests for (a, b) hosts entering the nest vicinity; (c, d) hosts approaching the model; and (e, f) hosts attacking the model, for each of the experimental model groups: male diederik cuckoo (red bar, dark grey line), female diederik cuckoo (grey bar, light grey line) and bulbul control (yellow bar, black line; a maximum of 24 hosts per treatment could respond to the simulated intrusions: one male and one female host per focal nest).

**FIGURE 3:**
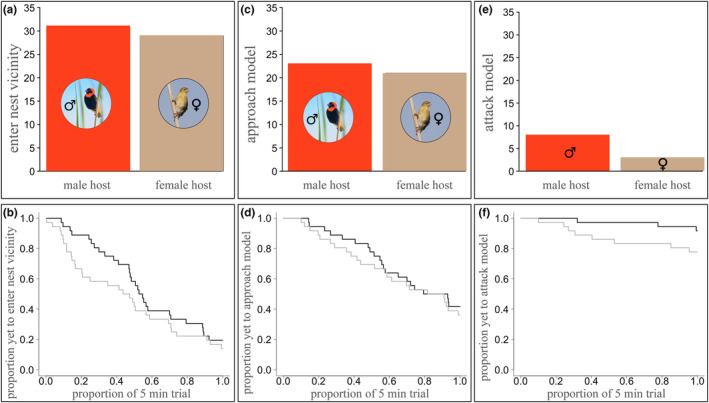
Frequencies of hosts that engage in frontline responses during the trial (top row) and their relative frontline response latencies (bottom row) during simulated intrusions of heterospecifics at hosts' nests for (a, b) entering the nest vicinity; (c, d) approaching the model closely; and (e, f) attacking the model, by the host male (red bar, dark grey line) and host female (beige bar, black line; a maximum of 72 hosts could respond to the simulated intrusions: 36 of each sex: one male and one female host parent per focal nest).

Most hosts (83%) entered the nest vicinity during the 5‐min model presentation, and 62% of hosts entered the nest vicinity and subsequently approached the model within approximately 40 cm during the trial period. Analyses of individual host responses to simulated intrusions at the nest revealed that the measures of model *detection* (entering the nest vicinity and approaching the nest) were similar across the three treatments. Treatment type did not have a significant effect on host probability (GLMM: *χ* ^2^ = 0.12, *p* = .94) and latency (MESM: *χ* ^2^ = 0.30, *p* = .86) to *enter the nest vicinity* (Table 1a, Figure 2a,b), neither did host sex (probability: *χ* ^2^ = 1.35, *p* = .24; latency: *χ* ^2^ = 1.84, *p* = .17; Figure 3a,b), or the interaction between treatment type and host sex (probability: *χ* ^2^ = 1.44, *p* = .49; latency: *χ* ^2^ = 0.33, *p* = .85). Similarly, treatment type did not have a significant effect on host probability (GLMM: *χ* ^2^ = 0.68, *p* = .71) or latency (MESM: *χ* ^2^ = 0.14, *p* = .93) to *approach the model* (Table 1b, Figure 2c,d), and again neither did host sex (probability: *χ* ^2^ = 0.31, *p* = .58; latency: *χ* ^2^ = 0.72, *p* = .40; Figure 3c,d), or the interaction between host sex and treatment (probability: *χ* ^2^ = 3.04, *p* = .22; latency: *χ* ^2^ = 2.64, *p* = .27).

To examine the possibility that host speed of approach varied according to treatment type, I also calculated the ‘approach window’ (lag in seconds between the time point at which hosts that did enter the nest vicinity then approached the model). There was also no significant effect of treatment on the approach window (LMM: *χ* ^2^ = 1.33, *p* = .51, *n* = 44 of 72 individuals; bulbul: *n* = 13; male diederik: *n* = 16; female diederik: *n* = 15), or the interaction between treatment and host sex (*χ* ^2^ = 0.53, *p* = .77), or host sex (*χ* ^2^ = 2.32, *p* = .13). Females that do enter the nest vicinity (*n* = 21) tend to approach the nest more rapidly (mean ± SE: 12.9 ± 4.41 s) than males (*n* = 23, mean ± SE: 49.4 ± 16.3 s). The mean absolute difference in approach window within a pair where each approaches the model (29 s; *n* = 16 pairs) was greater than between the mean male (26 s) and mean female (11 s) approach window across pairs (15 s; *n* = 16 pairs). The coefficient of variation across males (2.34), females (1.24), and the mean within‐pairs (0.80) shows a high degree of dispersion around the mean in each case.

### 3.2 Do male or female bishop hosts discriminate between male and female diederik cuckoo according to the direct threat‐level they pose to offspring?

While most hosts approached the model during the trial (75% of those that entered the nest vicinity), a smaller proportion (24% of individuals that approached the model) physically attacked the model by striking it with their beaks and/or feet (Figure 2 and 3). Where attacks on the model did occur, they were typically forceful and, in some cases, dislodged the model from the nest entrance, despite it being firmly attached to the supporting reeds. Occasionally, intense attacks recruited contributions from neighbouring males (from non‐experimental territories, 19% (7) trials), and these occurred evenly across space and across the experimental period. Due to the relative infrequency of such collective mobbing responses, it is not possible to make inferences about factors that contribute to their occurrence, but mobbing responses arose similarly across treatment groups (male diederik: 2, female diederik: 3 and bulbul: 2), so there is no indication that collective responses arise due to model discrimination. Indeed, analyses of individual behavioural responses revealed no significant effect of treatment type on attack probability (GLMM: *χ* ^2^ = 0.01, *n* = 72, *p* = .99; Table 1c) or latency to attack (MESM: *χ* ^2^ = 0.054, *n* = 72, *p* = .97, Figure 2e,f), and no significant effect of an interaction between treatment type and host sex (probability: *χ* ^2^ = 0.13, *n* = 72, *p* = .94, Table 1c; latency: *χ* ^2^ = 2.47, *n* = 72, *p* = .29), despite a significant effect of host sex on both the probability (*χ* ^2^ = 22.78, *n* = 72, *p* < .001, Table 1c) and the latency (*χ* ^2^ = 7.35, *n* = 72, *p* = .0067; Figure 3e,f) to attack the model. This effect is driven by male hosts carrying out the majority of attacks on the models, with females engaging less than half as frequently, and when females do attack, it takes them longer to do so (*n* = 3; mean 209 s) compared to males (*n* = 8; mean = 137 s). Because female hosts attack rarely and exclusively attack when the male host engages in attacking, a large variance is attributed to ‘nest ID’. Absolute estimates from this model should be treated with caution due to zero‐inflation that arises from attacks being rare (Figure 2e).

**FIGURE 4:**
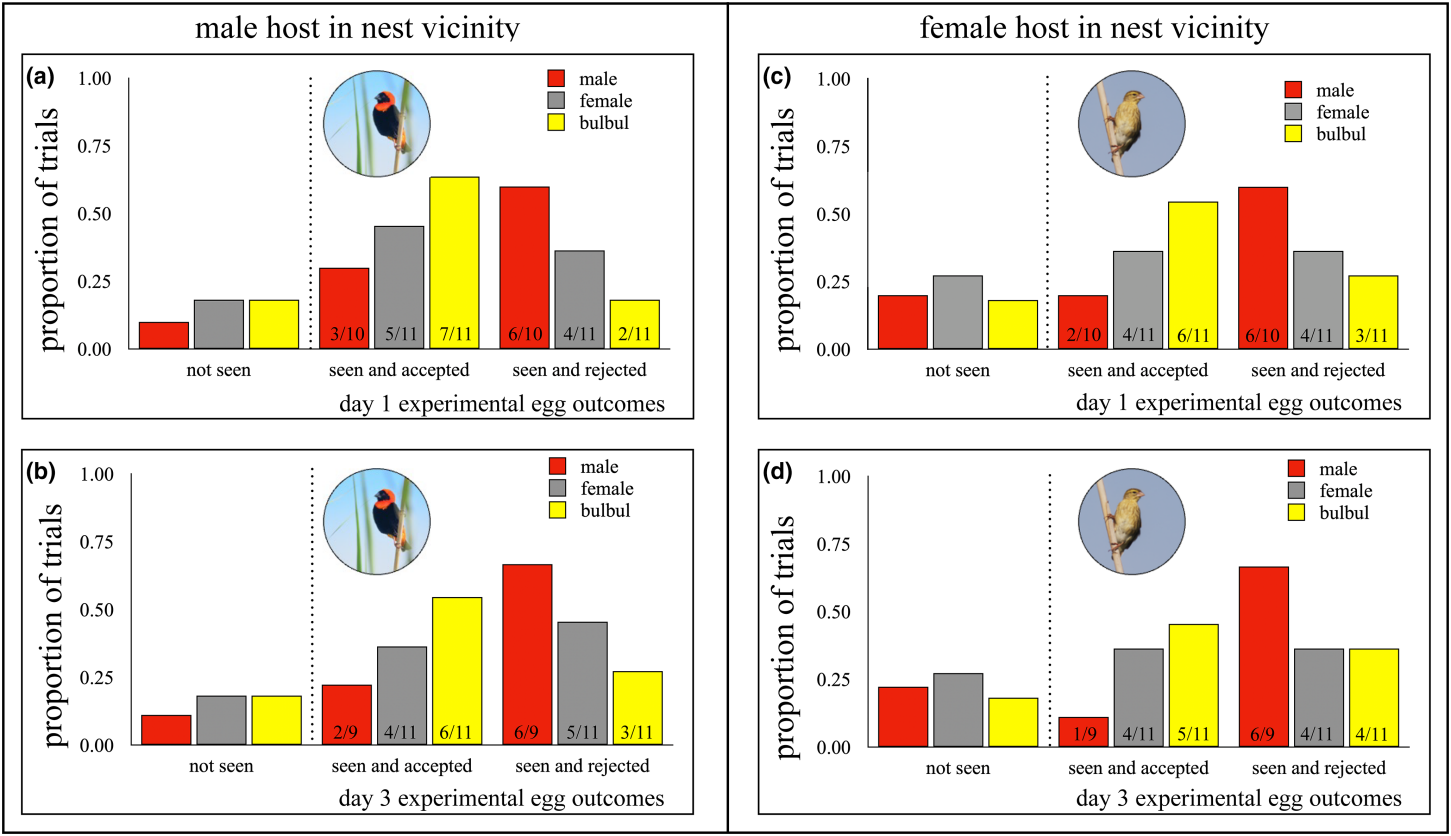
Responses to experimental brood parasitism at 1 day (top row) and 3 days (bottom row) according to whether the male host (a, b) or the female host (c, d) entered the nest vicinity during simulated heterospecific intrusions at hosts' nests, for each of the experimental model groups: male diederik cuckoo (red), female diederik cuckoo (grey) and bulbul control (yellow).

We do not yet know whether male, female or both parent hosts are responsible for egg rejection decisions in bishops, hence the analyses and contrasts below show both male and female scenarios. However, it is reasonable to assume that female hosts take the lead in egg rejection behaviours because males do not contribute to incubation or early life provisioning of nestlings in this species, so it is more likely that females reject eggs during post‐trial incubation (Friedl, 2004). Nevertheless, egg rejection estimates are provided for both male and female hosts at 1 and 3 days post trial, providing accumulative estimates at each time point. Male hosts entered the nest vicinity during over 80% of trials, while female hosts were 5% less likely than males to enter the nest vicinity during the trial. Egg rejection responses were largely similar after 1 day (GLM: *χ* ^2^ = 7.03, *n* = 32, *p* = .030) and 3 days (*χ* ^2^ = 6.76, *n* = 31, *p* = .034) since the trial, revealing a significant interaction between treatment type and whether the male host was in the nest vicinity during the trial (Table 2, Figure 4). There was also a significant interaction between treatment type and whether the female host was in the vicinity during the trial by day three (*χ* ^2^ = 9.05, *n* = 31, *p* = .011; Table 3, Figure 4c,d). When hosts are not in the nest vicinity during the trial, they are equally likely to accept or reject experimental eggs in each treatment group, whether it is a male or female host that views the model at the nest during the trial. Statistical contrasts between the treatment levels (male diederik–female diederik, male diederik–bulbul, female diederik–bulbul) are provided below to aid the interpretation of which treatment level(s) drive the significant interactions (Tables 2 and 3).

Overall, the higher probability of egg rejection after witnessing a male diederik cuckoo on the nest compared to the other two treatments explains the significant interaction effect in the main analyses, whether it is the male or female host that witnesses the nest intruder. When female hosts observe a male diederik cuckoo on the nest (*n* = 9), hosts reject significantly more experimental eggs by day three than when they are exposed to a female diederik cuckoo on the nest (*χ* ^2^ = 8.99, *n* = 11; *p* = .011; Table 3, Figure 4d). Hosts also have a higher probability of rejecting experimental eggs when a female host observes a male diederik cuckoo (*n* = 9) compared to a bulbul control model on the nest, but this was marginally non‐significant (*χ* ^2^ = 4.79, *n* = 11; *p* = .091; Table 3, Figure 4d). By contrast, when female hosts observe a female diederik model on their nest, hosts do not differentially reject experimental eggs (*χ* ^2^ = 2.28, *n* = 11; *p* = .32; Table 3, Figure 4d) when contrasted with hosts exposed to bulbul controls (*n* = 11). Consequently, the higher probability of egg rejection responses after witnessing a male diederik cuckoo on the nest explains the significant interaction effect in the analysis (Table 3, Figure 4d).

When male hosts observe a male diederik cuckoo compared to a female diederik cuckoo on the nest, hosts are not significantly more likely to reject experimental eggs at one (*χ* ^2^ = 4.73, *p* = .094) or 3 days post‐trial (*χ* ^2^ = 4.53, *p* = .10). Conversely, when a male host was in the nest vicinity during the trial, hosts rejected two‐thirds as many experimental eggs when exposed to a male diederik cuckoo (Day 1: 60% rejected; GLM: *χ* ^2^ = 7.56, *n* = 10, *p* = .023; Day 3: 67% rejected; *χ* ^2^ = 6.87, *n* = 9, *p* = .032; Table 2, Figure 4a,b) compared to hosts exposed to a bulbul control (Day 1–3: 18–27% rejected, *n* = 11). When male hosts observe a female diederik model on their nest, hosts do not differentially reject experimental eggs (Day 1: 36% rejected, *χ* ^2^ = 1.01, *n* = 11, *p* = .60; Day 3: 45% rejected, *χ* ^2^ = 0.91, *p* = .64) when contrasted with hosts exposed to bulbul controls (*n* = 11). Taken together, these contrasts suggest that the higher probability of egg rejection on exposure to a male diederik cuckoo drives the significant interactions in the main analyses, whether it is the male or female host that witnesses the nest intruder.

## 4 DISCUSSION

If female colouration in brood parasitic diederik cuckoos evolved to reduce detection by hosts at the nest, then hosts should be less likely, or take longer, to detect female diederik cuckoos compared to more conspicuous males and controls. I found no difference in the probability of hosts entering the nest vicinity or in hosts approaching the model, and there was also no difference in approach speed. The measures of stimuli detection used here are typical in ecology, taken across the possible window of detection, and none of the evidence suggests a difference in model detectability (for contrast, see effective crypsis: Koskepato et al., 2020; Troscianko et al., 2016). Taken together, my findings do not support the hypothesis that cryptic colouration of female diederik cuckoos makes them less detectable at the nest. However, female diederik cuckoos may nevertheless benefit from their relative anonymity compared to the more conspicuous appearance of male diederik cuckoos, since brood parasitism attempts are less likely to be successful when hosts observe a conspicuous male diederik cuckoo at the nest.

Why are bishop hosts more likely to reject eggs when they view a male diederik cuckoo model on their nest? This finding was the opposite of predictions, given that the female diederik cuckoo was the only treatment that presents a direct brood parasitism threat. This finding does not arise because females are less detectable (Figure 2a–c), or because hosts are less likely to approach closely enough to have the opportunity to identify salient features of female diederik cuckoos (Figure 2c). Instead, differential rejection responses could be explained by the high stimulus salience of a male diederik cuckoo at the nest, because hosts identify conspicuous male diederik cuckoos as an enemy, and may associate his conspicuous characteristics with a threat to their clutch. One component of male diederik cuckoo appearance that could be particularly salient to hosts is their red iris and eye‐ring (Figure 1c). While this may seem a relatively small component of diederik cuckoo overall appearance, avian eyes can be highly salient and important mediators of inter‐specific interactions (Davidson et al., 2014, 2017; Trnka et al., 2012). Furthermore, bishops make use of red colouration as a dominant component in their breeding plumage and their sensitivity to detecting and processing red signals is central to reproduction, therefore likely consequences of ‘sensory drive’ due to sensory biases from sexual signalling of hosts could explain their overall stronger rejection responses to witnessing a male diederik cuckoo at the nest (Edler & Friedl, 2010; Endler, 1992; Endler & Basolo, 1998). Given host egg rejection responses when faced with a male diederik cuckoo, it is certainly beneficial for female diederik cuckoos to be relatively anonymous, and it is notable that they lack a conspicuous red iris and eye‐ring (Reed, 1968). Indeed, simulated female diederik cuckoo nest intrusions provoke egg rejection responses to a similar extent as a harmless bulbul. While further examination of bishop perception would be necessary to confirm that diederik cuckoo red stimuli drive behavioural responses, it is relevant to note that the bulbul models included a conspicuous bright yellow patch, and so it is unlikely that my findings are driven simply by conspicuousness against the green‐brown background environment, since hosts were no more likely to reject experimental eggs on exposure to bulbuls (with a conspicuous yellow vent patch) compared to female diederik cuckoos (no conspicuous patch).

Could host responses to male diederik cuckoos be advantageous for brood parasitism? One conceivable selective benefit of male diederik cuckoo appearance tapping into the perceptual biases of bishops is that hosts could direct their mobbing efforts towards the more apparent threat of the male diederik cuckoo, which in turn, could generate or facilitate opportunities for secretive and rapid laying visits by females. Indeed, there are descriptions that suggest male diederik cuckoos, and other species of cuckoo, assist ovipositing females by distracting hosts away from the nest (see Friedmann, 1948, p. 162 and for further context: Davies, 2000; Jensen & Jensen, 1969; Rowan, 1983). However, I found no evidence that bishop hosts were more likely to attack, or attacked male diederik cuckoos more rapidly, compared to the other heterospecific intruders. Moreover, even if hosts are distracted with the task of aggressively repelling a male diederik cuckoo, thereby facilitating a window of opportunity for a stealthy female diederik cuckoo to lay undetected, those hosts that have observed a male diederik cuckoo in the nest vicinity are more likely to reject odd eggs in the clutch. Crucially, although it seems intuitive that hosts in an aggressive state provoked by the males’ red eye colouration (potentially due to sensory bias consequences of host sexual signalling; Ryan, 1990) subsequently reject odd eggs, there was no evidence that hosts were more aggressive when exposed to male diederik cuckoo compared to other heterospecific intruders. Interestingly, across Cuculini hosts, frontline aggression and egg rejection responses rarely correlate, suggesting that frontline behaviours (i.e. aggressive or wary responses) do not predict egg rejection decisions (Thorogood & Davies, 2016; York & Davies, 2017). Hosts may simultaneously find the red eye‐ring salient, and this may influence rejection decisions, while overt aggressive responses are variable across hosts. Given the findings presented here, diederik cuckoos would benefit from males avoiding close proximity to bishop nests, especially when female cuckoos are laying, and in particular from having females that do not look like males. Otherwise, if diederik cuckoo males approach the nest closely while accompanying more secretive laying female diederik cuckoos, the conspicuous male diederik cuckoos could provide a reliable indicator of brood parasitism risk for hosts and it could be costly when male diederik are observed in the nest vicinity by hosts.

There are two components of diederik cuckoo colouration that were not simulated with the models used in this study: reflectance in the ultraviolet (UV) wavelengths of the spectrum and plumage iridescence. Nevertheless, these components are unlikely to explain the findings reported here, or qualitatively alter interpretations. First, whether iridescent plumage might influence host responses to heterospecific intruders at their nest is highly unlikely since the iridescence of diederik cuckoos does not show strongly in the light environment where their hosts nest, but it could serve to further enhance diederik male conspicuousness when perched in direct sunlight (Reed, 1968). Second, although UV signalling is an important component in the plumage reflectance of many avian species and was long overlooked due to human inability to perceive dimorphic variation in wavelength reflectance in this range (Andersson et al., 1998), it is unlikely that diederik cuckoo plumage has extensive UV reflectance. A study of the short‐wavelength‐sensitive type 1 (SWS1) opsin gene indicates that shining cuckoos of this genus (*Chrysococcyx*) have violet sensitive (VS) and not ultraviolet sensitive (UVS) cones, which means colour vision selected for UV sensitivity (and UV signalling) is highly unlikely in diederik cuckoos (Aidala et al., 2012). Furthermore, feather analyses from common cuckoo (*Cuculus canorus*) did not reveal notable reflectance in the UV range which suggests UV reflectance is at least not a widespread characteristic of Cuculini cuckoo plumage (Koleček et al., 2019; Mullen & Pohland, 2008). Even if diederik cuckoo do exhibit UV signalling, it is likely the male would display greater UV reflective plumage than the female, since this is typical in avian UV sexual dimorphism (Andersson et al., 1998). If this were true, it would mean the estimates of host responses to simulated males are conservative since UV plumage reflectance might enhance their conspicuousness against the reed background, but nonetheless, unlike their red colouration, there is no *a priori* reason to predict that UV colouration in diederik cuckoos would be particularly salient to bishop hosts since bishops exhibit carotenoid‐based and not UV‐based plumage signalling (Edler & Friedl, 2010). Further work is now needed to investigate perception in host responses to diederik colouration.

Other than the effect of host sex (host males are more aggressive than females; Figure 3e,f), it is not clear what underlies aggressive response thresholds in bishops. Bishops are polygynous and males dominate aggressive responses towards nest intruders. Their threshold for engaging in an attack may be relatively high since they have numerous nests to defend, and studies of their breeding biology suggest economic and temporal trade‐offs in attacks against conspecific and heterospecific intruders could be possible (Edler et al., 2011; Metz et al., 2009). For example, male bishops spend considerable time and effort in nest construction and courtship display to attract multiple mates, and nest defence could trade‐off against these important tasks, which could dilute male investment in attacks. Whether or not bishop aggressive defences towards heterospecifics at their nests involve only generalised nest defences, or whether they possess diederik cuckoo‐specific defences was not examined here, but seems unlikely given the indiscriminate aggression towards all simulated heterospecific intruder categories, which contrasts with other hosts of brood parasitic Cuculini (Duckworth, 1991). While the relative infrequency of individuals engaging in attacks could be a limiting factor for detecting treatment effects in this study, it is worth noting that there was sufficient power to detect a sex difference in host aggression (Figures 2e,f and 3e,f). Further work to investigate how nesting density and colony size affect aggression thresholds and collective defence in bishops would be helpful for estimating population level variation in aggressive defence to which diederik cuckoos are exposed (Ferguson, 1994; Lawes & Kirkman, 1996).

The extent of variation in the aggressive behaviour of bishop hosts towards heterospecific intruders characterised in this population has three main implications for coevolutionary interactions and the evolution of sexual dimorphism in diederik cuckoos. First, despite approaching the model and looking directly at the model, a larger proportion of hosts did not then engage in an aggressive response. This highlights that diederik cuckoos visiting host nests may infrequently experience the direct costs of physical aggression from bishop hosts (because few hosts engage in attacking the model) or the increased vigilance of neighbours alerted to brood parasitism risk that is observed in other brood parasite systems (because aggression that recruits bishop neighbours occurs rarely; for contrast see: Campobello & Sealy, 2018; Thorogood & Davies, 2016). Nevertheless, this heterogeneity in host aggressiveness means both defences are an unpredictable risk associated with approaching host nests. Second, despite lacking prominent chest‐barring, diederik cuckoos do exhibit underwing barring that can be revealed flexibly, so further investigation is now required to determine whether host aggression towards diederik cuckoos is modulated by this concealed hawk‐like characteristic (Lyon & Gilbert, 2013; York, 2021). Finally, it is perhaps surprising that hosts were not more aggressive towards diederik cuckoos, given their reputation for fierce attacks upon diederik cuckoo (Rowan, 1983). However, it is important to recognise that such attacks are eye‐catching and even keen observers are unlikely to document instances where cuckoos are not attacked by hosts, which underlines the necessity for carefully designed experiments.

What are the consequences of female diederik cuckoo colouration in the context of cuckoo‐host dynamics at nests? Atypically for passerines, weaverbirds such as bishops begin egg laying before the nest is complete, and consequently, the eggs are visible through the weave of the nest (Davies, 2000). This fact could explain why, despite the potential costs of visiting nests, female diederik cuckoos may benefit from approaching nests closely since when coupled with brief laying windows (mode clutch size 3) and dense colonies with limited vantage points, it may be more challenging for her to monitor opportunities from afar. It is conceivable that if the ancestral state was for both male and female to exhibit showy red facial colouration, there would be a selective advantage for female facial colouration to become less showy to avoid costly conspicuousness while approaching nests. This sequence is supported by comparative analyses that reconstruct the most likely evolutionary pathway for sexual dimorphism in cuckoos involving a transition from showy to cryptic (Krüger et al., 2007). Furthermore, it is worth noting that, while red eye‐ring and iris colouration are unique among the African members of the genus *Chrysococcyx*, red facial colouration does occur in close relatives such as the Asian *C. xanthorynchus* and *C. maculatus*, Australian *C. minutillu*s, and elsewhere in the Cuculidae (e.g. parental Malkohas where both sexes exhibit showy red facial colouration, and more extensively than their male brood parasitic relatives).

These findings have several implications for the evolution and maintenance of sexual dimorphism in diederik cuckoos and across the Cuculidae. First, they provide new evidence that suggests sexual dimorphism in brood parasitic cuckoos has evolved and/or is maintained due to benefits in coevolutionary interactions with hosts. However, these findings lead me to suggest an alternative perceptual mechanism to females simply avoiding detection via cryptic appearance. While females appear more cryptic and males are more conspicuous, there was no difference in how detectable or how likely hosts were to approach male or female diederik cuckoos at the nest, relative to controls. This does not necessarily mean that the more cryptic appearance of females is not beneficial in reducing detection in all contexts (e.g. females may avoid harassment while monitoring host nests from afar), but given the egg rejection costs of male‐like appearance, and given that there is no evidence to confirm that cryptic appearance hampers detection by hosts of diederik cuckoo, avoiding detection at the nest via appearance may be less important than the effect of being less salient to hosts if the cuckoo *is* witnessed in the nest vicinity. It could therefore be illuminating for us to specify two mechanisms for traits associated with female cuckoo phenotypes that may function to evade host defences: those that are cryptic (avoid detection) and those that are anonymous (not salient to hosts). Second, given that salience may underlie how host perceptual mechanisms influence selection on adult cuckoo appearance, we might expect to find host species‐specific effects, particularly in those that lack red colouration. In these cases, other cuckoo traits and behaviours might be more important and could co‐occur with host‐specific gentes (Gibbs et al., 1996; Jensen & Vernon, 1970; Reed, 1968). It also remains possible that showy traits in some male brood parasitic cuckoos could be beneficial in coevolutionary interactions with hosts or could even be *synergistic* with the evolution of cryptic or anonymous traits associated with female phenotypes, but at least in diederik cuckoo, hosts appear to perceive conspicuous males as a brood parasitic enemy and his presence near the nest decreases brood parasitism success. Finally, given that in some species of brood parasitic cuckoos facial colouration occurs as distinct sex‐specific morphs (diederik cuckoo), and in other species female polymorphisms have benefits in brood parasitic interactions with hosts (common cuckoo), the role of host perception and defences against brood parasites may be much more important than sexual selection in the evolution of sex‐specific morphs within this group (Kruger et al., 2007; Mank, 2023; Thorogood & Davies, 2012). Nonetheless, there is some evidence of multifunctional behavioural signalling in adult brood parasitic cuckoos (Moskát & Hauber, 2019), and so further research on the role of sexual selection and mating systems in the evolution of adult cuckoo phenotypes will aid a complete understanding of these complex and multimodal suites of traits.

## 5 CONCLUSIONS

Across cuckoos, brood parasitic females are more cryptic than males (Krüger et al., 2007), yet the benefit of cryptic plumage, in diederik cuckoos at least, is not clearly linked to the benefits of avoiding detection by hosts, since there is no evidence that bishop hosts differentially detect heterospecifics at the nest. Despite the fact that the vast majority of hosts (83%) entered the nest vicinity and subsequently closely approached the model, the likelihood and speed at which hosts approached the model were nearly identical across treatment groups. However, female diederik cuckoos may nevertheless benefit from the relative anonymity that their appearance bestows compared to the more conspicuous appearance of male diederik cuckoos, since bishop hosts differentially reject experimental eggs when female hosts observe a male diederik cuckoo at the nest. This finding is the complete opposite of predictions if hosts discriminate between male and female diederik cuckoo on the basis of their brood parasitism threat‐level, which would allow hosts to perform responses adjusted to the sex‐specific level of the threat. Furthermore, bishop hosts were indiscriminately aggressive towards simulated heterospecific intruders at the nest. Together, these findings suggest that, despite bishop hosts having the capacity to mount frontline mobbing and egg rejection defences, they remain vulnerable to brood parasitism by diederik cuckoos because it is challenging for them to correctly identify and respond appropriately to the threat, with hosts more likely to reject experimental eggs after viewing a conspicuous male diederik cuckoo. Consequently, host perceptual processes may explain why diederik cuckoo sexual dimorphism is characterised by a more anonymous female that has diverged from a male with specific conspicuous characteristics, but further investigation is needed to determine the specific stimuli that provoke host responses. Combined, the untuned defences of bishop hosts maintain their susceptibility to brood parasitism by sexually dimorphic diederik cuckoos. While the cryptic appearance and behaviours of female cuckoos may facilitate evading host detection in some contexts, my findings suggest that the costs of conspicuous male‐like appearance could drive the benefits of sexual dimorphism in the colouration of brood parasitic cuckoos. These findings therefore indicate important factors and pathways that may underlie the origin and maintenance of sexual dimorphism across the Cuculidae.

## AUTHOR CONTRIBUTIONS

**Jennifer York:** Conceptualization (equal); data curation (equal); formal analysis (equal); funding acquisition (equal); investigation (equal); methodology (equal); project administration (equal).

## FUNDING INFORMATION

These contents do not reflect the views of the University of Cambridge or the European Commission. This study was funded by a Research Grant from the Association for the study of Animal Behaviour and an Individual Fellowship (Global) from the European Union's Horizon (2020) Research and Innovation Program (Marie Skłodowska‐Curie IF Grant Agreement no. 837838) to J.E.Y.

## CONFLICT OF INTEREST STATEMENT

None.

## Data Availability

Supporting data and code are available at datadryad.org: https://doi.org/10.5061/dryad.g79cnp5xx.
